# Experiencing a slow passage of time was an indicator of social and temporal disorientation during the Covid-19 pandemic

**DOI:** 10.1038/s41598-022-25194-2

**Published:** 2022-12-26

**Authors:** Pablo Fernandez Velasco, Bastien Perroy, Umer Gurchani, Roberto Casati

**Affiliations:** 1 Trinity College Dublin, Dublin, Ireland; 2 Institut Jean Nicod (ENS, EHESS, CNRS), Paris, France

**Keywords:** Human behaviour, Epidemiology

## Abstract

Time dilation was experienced in most countries and across the several years of the Covid-19 crisis: the passage of time was deemed slower than before the pandemic, and the distance to the beginning of the pandemic seemed longer than it really was. An outstanding question is how these two aspects of time judgements relate to other temporal, social and affective disturbances. We developed and validated a 59-item questionnaire to explore these questions. 3306 participants completed the questionnaire in France in May and June 2021. Here, we analyse group differences and find that both slow passage of time and long distance judgements were associated with larger disturbances across all domains under study. These included temporal disruptions—the aptness to project oneself into the future, the sense of a rift between pre-pandemic and pandemic time, the ability to locate oneself in time, the capacity to recall the order of past events—, as well as an overall sense of social disorientation, and trauma-specific disturbances. In contrast, both fast passage of time and short distance judgements were associated with beneficial effects across all of the mentioned domains. Our results indicate that perceived passage of time and temporal distance judgements are key indicators of social and temporal disorientation.

## Introduction

As a result of the Covid-19 crisis and its associated public health measures, many people have suffered a host of disruptions in their perception of time. To capture the heterogeneity of these disruptions, we developed a quantitative questionnaire with 59 questions. Here, we analyse 3306 responses to the questionnaire with the aim of elucidating the way in which disturbances to the feeling of the passage of time and to the sense of temporal distance relate to other types of temporal, social, and affective disruptions, such as the capacity to remember the order of past events or the difficulty in imagining future events. By studying the psychological impact of the pandemic, we gain insight into how disruptions in different aspects of time judgements relate to each other.

Abnormal temporal experience has been a persistent feature throughout the Covid-19 crisis^1–9^, and, consequently, there is a multitude of studies focusing on different types of time distortions. Most of the existing research on time judgements during the pandemic has focused on *passage of time judgements*—the sense of time passing faster or slower. Previous studies have found a pervasive sense of time passing slower than usual in France^1,10^, Italy^7^, and Iraq^5^, a pervasive sense of time passing faster in Uruguay^8^ and Argentina^6^, and both fast and slow passage of time in the United Kingdom^2,11^. The differing directions of the experienced distortions from one country to another and from one study to another are partly due to cultural and demographic aspects. The researchers studying time judgements in Iraq highlight the local polychronic time culture, and the study in Argentina found significant gender differences. However, these two elements alone do not fully account for the diversity found in the reported disruptions. Other factors include the time at which the questionnaire was asked (e.g., during or after a lockdown), and whether participants reported a present or a retrospective judgement^12^, and if retrospective, whether it was over a short or a long period^1,10^.

A second key aspect of time judgements corresponds to *subjective temporal distances*^2,4,5,9,13^. Temporal distance judgements express the subject’s sense of the length of time having elapsed between a given past event and the present moment at the time of questioning (e.g. how far away the beginning of the pandemic seems to the subject). While some studies refer to it as (depending on the distance under evaluation), *8-months passage of time judgments*^2^, or *11-months passage of time judgments*^5^, the common phenomenon under study is the short-to-long feeling aspect of time. To clarify, temporal distance judgements are not the same as retrospective passage of time judgements, which are feelings of the passage of time over a long period (e.g. “since the pandemic began, time has been passing slowly”). Therefore, expressions such as “*11-months passage of time judgments”*, are to some extent, a misnomer, because the actual question employed in the study using that term was not about the passage of time (e.g. fast or slow) over an 11-month period, but about the feeling of elapsed time: “It is 11 months since Iraq first went into lockdown. It feels like (1) a lot shorter, (2) somewhat shorter, (3) about 11 months, (4) somewhat longer, (5) a lot longer.” Consequently, it is important to draw a distinction between passage of time judgements (which can be present or retrospective) and temporal distance judgements.

Compared to passage of time judgements, temporal distance judgements are hypothesised to rely more heavily on memory processes^2,14^. Temporal distance judgements might also be influenced by the vividness of the event at the beginning of the period in question^15^; in the case under consideration, the beginning of the pandemic. Most studies have found that distance judgements corresponded to pandemic time being longer than pre-pandemic time, which was associated with slower passage of time^2,9^. In contrast, a study in Germany compared passage of time data during the pandemic to data preceding the pandemic, and found an overall relative deceleration in the experienced passage of time in between these two points^4^. But the felt passage of time was still deemed overall fast, which aligned with an overall short experience of pandemic time. Moreover, the study found that the perceived subjective temporal distance of pandemic time showed a bimodal distribution, with a majority of participants reporting a shorter time length but a minority of participants reporting a longer one. All in all, it seems that in general, if time is experienced as slow, it is also experienced as elongated, and if it is experienced as fast, it is also experienced as compressed.

Table 1 summarizes all Covid-19 empirical studies to date assessing either passage of time or temporal distance judgements, and outlines divergences in methodological strategy. Some researchers asked for a judgement for the passage of time about before the pandemic, and then during the pandemic, and compared both^1,10,16^. A wealth of international studies asked for judgements relative to the time before the pandemic and analysed responses directly^2,5,6,11^. A single study compared time judgements expressed before the pandemic and those expressed during the pandemic^4^. A study investigated *time expansion*^3^ by focusing on a host of phenomenological features previously validated to be indicative of the passage of time. By contrast, while studies looking at temporal distance judgements differed in the name they gave to the judgement, they actually all used a similar frame for it. Our own study framed both passage of time and temporal distance judgements not with the goal of comparing the Covid judgements with the pre-Covid ones, but rather to allow for conflicting feelings about the pandemic (e.g. both a *faster* and *slower* feeling of passage of time, *at times*) to be expressed. Our way of framing passage of time judgements is similar to the study from Loose et al.^8^, but their study didn’t focus on bimodality and didn’t investigate distance judgements. Shared emphasis is put on the association between the slow passage of time and boredom^1,2,4^, depression or psychological distress^2,8,16^, perceived isolation or an overall decrease of satisfaction in terms of social interactions^4,9,11^. As the slow passage of time is usually associated with longer temporal distances, the abovementioned emphasis was also expressed for long temporal distance judgements, when investigated^2,9,13^.

**Table 1:** A summary of existing passage of time and temporal distance judgements studies related to the Covid-19 crisis.

Study	Judg.	Frame 1	Frame 2	Frame 3	Frame 4	Frame 5	Modal result	Bimodal	Completion time
Martinelli et al.^16^	PoT	How do you feel about the speed of the passage of time now?	How do you feel about the speed of the passage of time during the lockdown?			How do you feel about the speed of the passage of time before the lockdown?	Slower (comparing before judgments with during lockdown judgments)	?	1st French lockdown, 2020
Droit-Volet et al.^1^	PoT	How do you feel about the speed of the passage of time now?	How do you feel about the speed of the passage of time during the lockdown?			How do you feel about the speed of the passage of time before the lockdown?	Slower (comparing before judgments with during lockdown judgments)	?	1st and 3rd French lockdowns, 2020 and 2021
Droit-Volet et al.^10^	PoT	What are your feelings about the speed of the passage of time now?	What are your feelings about the speed of the passage of time for the day?	What are your feelings about the speed of the passage of time for the week?		What are your feelings about the speed of the passage of time before the lockdown?	Slower (comparing before with during lockdown)	?	1st French lockdown, 2020
Ogden^11^	PoT		Thinking about today, how quickly has time felt like it is passing in comparison normal (i.e. before lockdown)?	Thinking about this week, how quickly has time felt like it was passing in comparison to normal (i.e. before lockdown)?			Both slower and faster	Y	1st UK lockdown, 2020
Ogden^2^	PoT		Thinking about today, how quickly has time felt like it is passing in comparison normal (i.e. before lockdown)?	Thinking about this week, how quickly has time felt like it was passing in comparison to normal (i.e. before lockdown)?			Both slower and faster	Y	2nd UK lockdown, 2020
Cellini et al.^7^	PoT	? (an item "that time is passing slowly")					Slower	?	1st Italian lockdown, 2020
Alatrany et al.^5^	PoT		Thinking about today, how quickly has time felt like it is passing in comparison with normal (i.e. before lockdown)?	Thinking about this week, how quickly has time felt like it was passing in comparison to normal (i.e. before lockdown)?			A little slower than normal	N	2nd Iraqi lockdown, 2021
Brenlla et al.^6^	PoT		“Thinking about today, how quickly time has felt like it is passing in comparison with normal (i.e. before lockdown)?”	Thinking about this week, how quickly has time felt like it was passing in comparison to normal (i.e. before lockdown)?			Somewhat faster than normal	N	3rd phase of the 1st Argentina lockdown, 2020
Chaumon et al.^9^	PoT			For you, these last few days have passed:			Slower	?	Longitudinal in 12 countries (Arg, Can, Col, Fr, Ger, Gre, Ind, Ita, Jap, Turk, the UK, the USA), 2020 and 2021
Cravo et al.^3^	PoT	My time seems empty	I often think that time just does not want to pass	I often feel bored	I have a lot of time	I often have spend time without doing anything	Expanded (slowing down)	?	First months of the pandemic in Brazil, 2020
Loose et al.^8^	PoT		My days pass more slowly, time extends	My days pass more quickly, time flies			Faster	?	Students during a no-lockdown situation in Uruguay but with schools closed
Kosak et al.^4^	PoT		When looking back, how did the last 12 months pass by for you?			[Asked before the pandemic] When looking back, how did the last 12 months pass by for you?	Slower (comparing pre-covid judgments with during-covid judgments)	Y	3rd wave in Germany, 2021
This study	PoT		At times, since the pandemic began, time has been passing noticeably slowly	At times, since the pandemic began, time has been passing noticeably quickly	Overall, since the pandemic began, time has been passing slowly/quickly		Slower	Y	End of the 3rd French lockdown, 2021
Ogden^2^	TD	It is 8 months since the UK first went into lockdown. It feels like…					A lot longer	Y	2nd UK lockdown, 2020
Alatrany et al.^5^	TD	It is 11 months since Iraq first went into lockdown. It feels like…					A lot longer	N	2nd Iraqi lockdown, 2021
Chaumon et al.^9^	TD	Take a a moment to think about a day a week from now. Days in the future can seem closer or further irrespective of when they will actually occur. This particular day seems to you:	Take a a moment to think about a day a month from now. Days in the future can seem closer or further irrespective of when they will actually occur. This particular day seems to you:	Take a moment to think about the FIRST day you started confinement. Past days can seem closer or further away than when they really happened. This particular day seems to you:			Longer	?	Longitudinal in 12 countries (Arg, Can, Col, Fr, Ger, Gre, Ind, Ita, Jap, Turk, the UK, the USA), 2020 & 2021
Kosak et al.^4^	TD	Since the beginning of the first lockdown due to the coronavirus here in Germany 14 months have passed. How long does this period feel?					Shorter	Y	3rd wave in Germany, 2021
Ogden and Piovesan^13^	TD	It is now 1 year since the beginning of the first national lockdown (23rd March 2020). How long ago does it feel for you?					Longer	Y	Step one in a four-step plan to remove all restrictions in the UK, 2021
This study	TD	At times, the beginning of the pandemic feels noticeably far away	At times, the beginning of the pandemic feels noticeably close	Overall, times before the pandemic feel as if they are further away/closer to me than they really are			Slower	Y	End of the 3rd French lockdown, 2021

When possible, similar frames (i.e. the scale given to respondents when probing their judgements) are grouped on the same columns.

Existing research has reported a variety of temporal disruptions that go beyond fast-slow passage of time and long-short distance judgements. These include disturbances in memory capacity (e.g. remembering intentions^17^), switches in temporal perspectives^8,18^, and difficulty in locating oneself temporally (knowing the present day of the week^7^). Overall, disturbances in time judgements are correlated with compliance with health mandates^19,20^, as well as with overall mental health^21^, in particular with regards to anxiety^17^, boredom^16,22^, and the feeling of isolation^2–4,9^. The current study aims to clarify the connection between different types of temporal disruptions. More specifically, the aim is to investigate how slow-fast passage of time and short-long temporal distances relate to other disturbances in temporal experience (e.g. difficulty in locating oneself in time). Previous studies found a bi-modal distribution for both passage of time and temporal distance judgements^2,4,11,13^. This means that across all aspects of the experience of the pandemic, whether time passed quickly or slowly was one of the most divisive phenomenological features, along with whether elapsed time felt shorter or longer. Moreover, a previous analysis of qualitative reports gathered during lockdowns and curfews in France and in the UK^23^ found that a substantial number of people reported that time passed both quickly and slowly *at once* depending on the perspective and temporal scale they had in mind, a confusion-inducing phenomenon which is conducive to *temporal vertigo*^5,13,24^. Such puzzling reports were widely documented during the pandemic^25^ and are investigated both for passage of time and temporal distance judgements^13^.

The exact nature of the distinction between fast and slow passage of time is still a topic of debate. Some have argued that “fast” judgements are actually inferences, e.g. about having had fun at a party and not noticing time flown by, while slow judgements are about actual experiences, e.g. a feeling of monotony waiting in a queue^26^. Others insist these judgements are tied to autobiographical memory activation: the fewer the events being remembered, the faster time passes^27^. Moreover, we must also take into account an additional distinction between present and retrospective passage of time judgements, which we mentioned earlier. A recent theory is that present passage of time judgements are based on contextual information^28^, while retrospective passage of time judgements depend upon other mechanisms, such as episodic memory. In any case, the consensus is that passage of time judgements are *not* explicit duration judgements (e.g. estimating it has been exactly 3 min since I received my last email notification)^29^. Passage of time judgements do not seek to represent time objectively, but rather to *express* something about a subject’s integration within her environment, in the characteristic fashion of metacognitive feelings^30^. Existing research indicates that arousal typically fastens the passage of time, while boredom and monitoring time through attention has the inverse effect^31^. All in all, whether the fast-to-slow spectrum of the passage of time judgements encompasses a real divide (e.g. if it were a mere mnemonic construction) or whether it is a plain continuous experiential feature (e.g. any event being possibly experienced *fast* or *slow*) remains to be investigated.

Our paper aims at providing new evidence regarding whether the fast/slow divide could be associated with experiential disturbances across various domains during the Covid-19 pandemic. To this end, we developed a quantitative questionnaire to capture different aspects of people’s experience, which we distributed in May and early June 2021. Participants responded to our questionnaire not during the first lockdown in 2020, but during the last phase of the 2021 French curfew, directly following the 3rd French lockdown. Thus, our focus is not exclusively on the impact of lockdowns, but rather on the protracted temporal disruptions of the pandemic, and on whether the feeling of slowing down of passage of time reported during the first lockdown persisted afterwards. Individuals who experienced a perduring slowing down of time may have adjustment difficulties related to mental health issues, and passage of time might thus serve as a predictor of psychological distress^32^.

As people's judgements about temporal distances (i.e. whether time appeared long or short) were concomitant with their judgements of passage of time (i.e. whether time passes fast or slow), we explore the short/long divide along with the fast/slow divide. Finally, we explore the impact of temporal vertigo, both in terms of passage of time (both fast and slow) and temporal distance (both long and short). Therefore, the present paper aims to examine the following three previously unexplored questions:

- Q1. Which group among those who report fast/slow feelings of the passage of time experienced stronger disturbances in the other aspects of temporal experience.
- Q2. Which group among those who report short versus long temporal distances experienced stronger disturbances in the other aspects of temporal experience.
- Q3. Whether temporal vertigo during Covid-19 was an aggravating factor of the overall time disturbances.

## Methods

We developed a quantitative questionnaire called ITSD (*Instrument for measuring Temporal and Social Disorientation*) through several stages^33^. First, we collected responses to an online, open-ended, qualitative questionnaire from 161 participants in March 2021. We conducted a thematic analysis of the temporal disruptions responses through a two-coder process. We then created the quantitative questionnaire based on the emerging themes from the analysis. We proceeded to send the quantitative instrument to four separate experts for feedback, which resulted in the modification of some questions, and in eliminating 12 questions (largely because experts suggested that a large number of questions might hamper participation). The resulting quantitative questionnaire was distributed in May and June 2021 through both email (4724 respondents) and Twitter (727 respondents). In France, the distribution period of the questionnaire corresponded to the final weeks of a 6-month-old curfew, which just followed the 7 weeks of the third national lockdown. There was no financial compensation for completing the questionnaire. The sample size was 5453 participants, of which 3306 participants completed the full survey comprising 59 questions, including 9 demographic questions, the MacArthur Scale of Subjective Social Status, 11 questions on lifestyle changes (pre- vs post-pandemic), 9 questions about social disruptions, 24 questions about temporal disruptions (divided into three blocks) and 13 questions adapted from the Global Psychotrauma Screening. (See Table 2 for sample characteristics.) The questionnaire was hosted on Qualtrics, was fully anonymous, and participants completed a written consent form online (sample characteristics are given in Table 1). All research involving human participants has followed the ethical procedures for approval at our institution. The Pôle Éthique of the Institut des Sciences Biologiques (INSB) of the Centre national de la recherche scientifique (CNRS) waived ethical approval for fully anonymous questionnaires. The study has been conducted according to the principles expressed in the Declaration of Helsinki.

**Table 2:** Sample characteristics of the 3306 participants having answered all questions.

Characteristics	Values
Average age	25.24
Median age	21.00
Number of female participants	2273
Number of male participants	990
Number of participants that chose neither ‘male’ nor ‘female’	43
Average socio-economic status (based on the MacArthur Scale of Subjective Social Status)	6.04
Number of students	2392
Number of workers	342
Number of retirees	64
Number of unemployed	25
Number of ‘chômage partiel’ (furlough)	3
Average number of cohabitants	2.3

The questionnaire was validated through the “3 faced construct validation method”^34^ using factor analysis. To achieve these three steps, the sample is randomly split into three parts, 20% for EFA, 40% for an CFA and 40% for a cross-validating CFA. Results of these validation steps are provided in the supplementary materials. This factor analysis revealed several unified components, each corresponding to a set of related questions. Table 3 contains Cronbach's alpha values for each component whereas other detailed results of factor analysis (e.g. factor loading table) can be found in the supplementary materials (Appendix 1). Questions about social disruptions formed a unified component (Social Disorientation; Questions 20, 21, 22, 23, 24, 27; e.g. “I feel more isolated from others”). The questions in Social Disorientation pertained to *perceived isolation*, *group belonging*, *closeness to others*, *the capacity to find emotional support*, and the overall sense that one’s social universe has been diminished. In contrast, temporal disruptions clustered into the following six separate components, which is a testament to the heterogeneity of temporal disruptions, as discussed in the introduction:

1. Passage of Time | Questions 48, 49, 50 | e.g. “Overall, since the pandemic began, time has been passing slowly/quickly”
2. Temporal Distance | Questions 45, 46, 47 | e.g. “At times, the beginning of the pandemic feels noticeably close.”
3. Temporal Order of Events | Questions 42, 43, 54 | e.g. “At times, I feel confused about the order of events that occurred since the pandemic began.”
4. Future Orientation | Questions 52, 56, 57 | e.g. “I feel it is easier/harder to imagine for me to imagine the future”
5. Temporal Self Location | Questions 40, 41 | e.g. “I get confused more/less often about which month of the year it is.”
6. Temporal Rupture | Questions 44, 58 | e.g. “At times, the period since the pandemic began felt unreal to me.”

**Table 3:** Cronbach's alpha values for components.

Construct	Cronbach’s alpha value
**Social disorientation factor**	
Social disorientation	0.865
**Temporal disorientation factors**	
Passage of time	0.80
Temporal order of events	0.63
Temporal distance	0.65
Future orientation	0.74
Temporal self location	0.65
Temporal rupture	0.41

It is worth noting that both passage of time (PoT) and temporal distance (TD) included three questions rather than one, which aimed at getting a better picture of potential bimodality. In the case of PoT, the first question is an agree-disagree scale querying for instances of slow time, the second an agree-disagree scale querying for instances of fast time, and the last question is a slow-fast scale for the overall passage of time during the pandemic. TD questions follow a similar structure. As we were expecting conflicting time feelings based on our preliminary qualitative reports, this design would allow us to shine a new light on the fast/slow and short/long divide, compared to other studies on the matter. *Future orientation* is the subject’s ability to imagine future events and the degree of associated anxiety. *Temporal rupture* corresponds to the feeling that there is a schism between the events preceding the pandemic and those that came after, together with a pervasive sense of time feeling unreal. *Temporal self-location* refers to the subject knowing what month of the year, or what day of the week, it currently is. Finally, *temporal order of events* corresponds to the ease with which a subject feels she is able to remember the order of past events. On top of the aforementioned components, questions 61–69, adapted from the Global Psychotrauma Screening^35^, formed a further component (hereon GPTS).

Apart from capturing ambivalent feelings about time passage and temporal distances, another advantage of our study comes from the large number of both questions and participants. Having 59 questions provides a fine grain understanding of disruptions in time judgements. A taxonomy of disruptions emerges out of the clustering of questions into components. We then calculated the values of each of the components in our survey. Values for each component were calculated by averaging the responses of each participant to the questions related to that component. In turn, the high number of participants means that we can divide the sample into smaller subgroups of participants to run comparative analyses all the while maintaining satisfactory statistical significance.

We decided to divide our sample into groups based on their time judgements during the pandemic for both our passage of time and temporal distance components. Below is the break-down of groups based on passage of time:

1. People who perceived that at times, since the beginning of the pandemic, time passed **faster**. (n = 801, or ≈ 24% of our sample)
2. People who perceived that at times, since the beginning of the pandemic, time passed **slower**. (n = 1681, or ≈ 51% of our sample)
3. People who perceived that at times, since the beginning of the pandemic, time passed **both faster and slower.** (n = 247, or ≈ 7% of our sample)
4. People who perceived that at times since the beginning of the pandemic, time passed **neither faster nor slower.** (n = 577, or ≈ 17% of our sample)

Below is the breakdown of groups based on temporal distance:

5. People who perceived that at times during the pandemic, its beginning seemed **far away.** (n = 2397, or ≈ 73% of our sample)
6. People who perceived that at times during the pandemic, its beginning seemed **close.** (n = 127, or ≈ 4% of our sample)
7. People who perceived that at times during the pandemic, its beginning seemed **both far away and close.** (n = 289, or ≈ 9% of our sample)
8. People who perceived that at times during the pandemic, its beginning seemed **neither far away nor close.** (n = 493, or ≈ 15% of our sample)

We wanted to allow for the potential of some subjects experiencing certain positive effects from the pandemic situation. This meant plotting responses on a disoriented-oriented spectrum, rather than on a disrupted–undisrupted spectrum. To this end, we rescaled each component between − 2 and + 2, with 0 meaning no experienced temporal or social disruptions. The disorientation-orientation axis offers a helpful framework through which to encompass the diverse disruptions across temporal, social and affective domains^36–43^. In contrast, the two components of interest related to time judgements functioned between slow versus fast time on the one hand, and long versus short time on the other, and couldn’t be a priori reduced plotted onto a disorientation-orientation spectrum. We excluded both PoT and TD from the analysis as they served as the pivot components that we investigate and make use of to divide our sample. The only component that does not span the full − 2 to + 2 scale is GPTS, which only probes one way towards trauma (for practical purposes, we deemed it a disorientation-like feature), so it is compressed in between the − 2 to 0 range. We calculated means and Cohen’s d values based on the above grouping. We then performed multiple one-way ANOVA tests to determine the statistical significance between the groups based on each pair of means.

## Results

**Figure 1:**
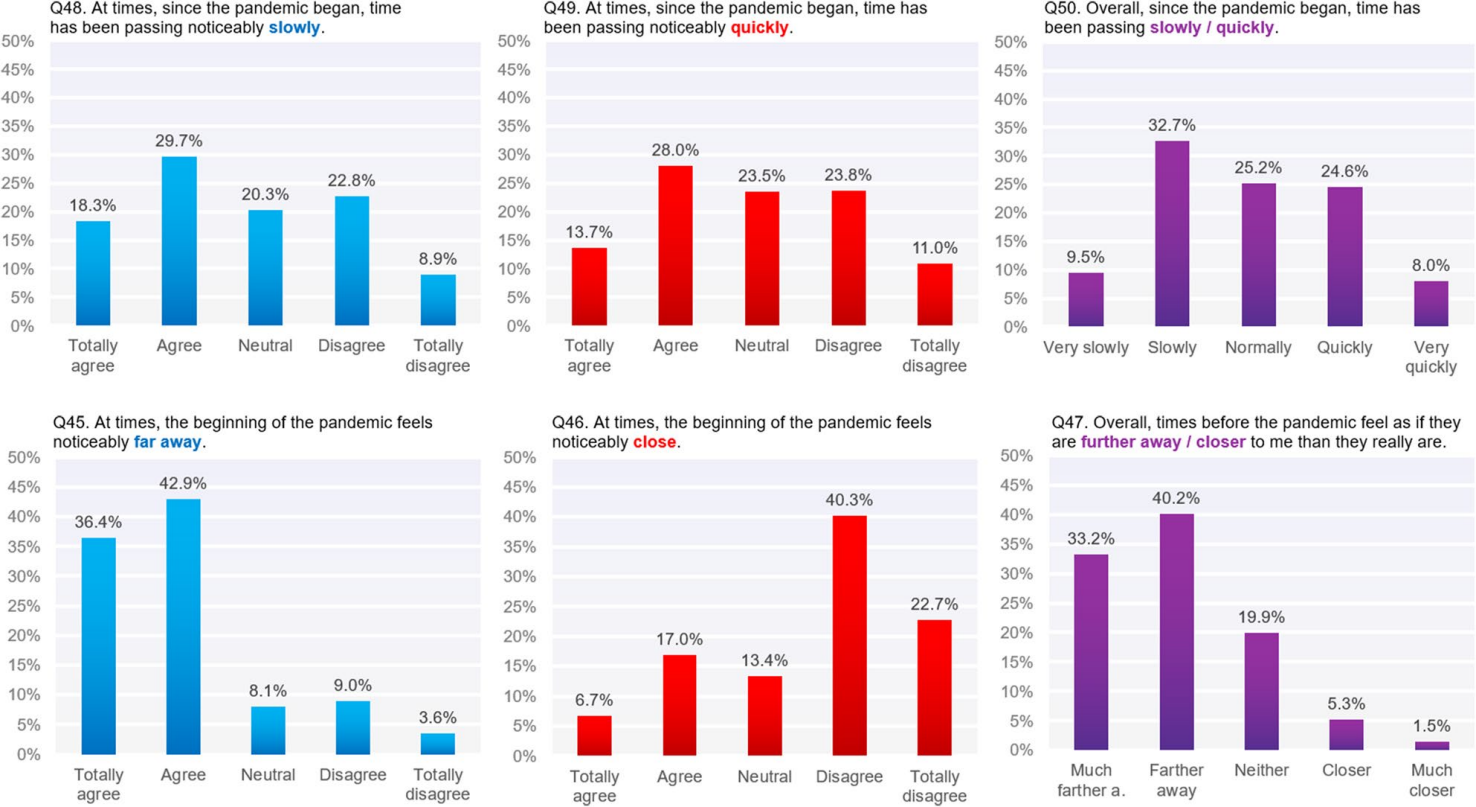
Distribution of PoT and TD questions within our sample.

Figure 1 shows the responses to the questions in the components PoT (*passage of time*) and TD (*temporal distance*), both of which show bimodality, even if the bi-modality of temporal distances judgement only clearly appears in the “at times” questions.

**Figure 2:**
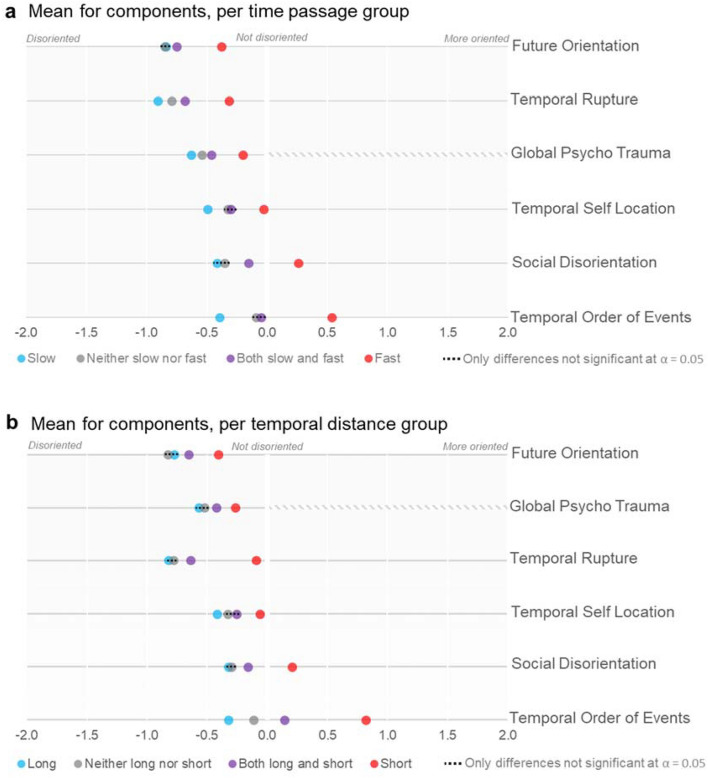
Mean components per time judgements groupings.

Figure 2 displays the 6 resulting spectrums for both the *passage of time* and the *temporal distance* groupings outlined in the methods section. For example, the top row in Fig. 2a shows there are significant differences in *future orientation* values between the group that experienced ‘fast passage of time’ and all other groups. The complete set of results for all components are presented in Fig. 2a, for PoT, and Fig. 2b, for TD. A total of 77% of differences between pairs of PoT and TD groups within all components were significant (α = 0.01). Only nine differences were not significant at the 0.05 α threshold and are represented with dotted lines in Fig. 2. Full statistics (Anova values, Cohen’s d values, means) are available in the supplementary materials (appendixes 3 and 4).

The largest differences in terms of Cohen’s d appear between those experiencing only fast and only slow time on the one hand, and those experiencing only short and only long time on the other. Within the passage of time groups, the largest difference for the fast group compared to the slow group is less trauma (F = 261.2; *p* < 0.01; d = 1.22), followed by less social disorientation (F = 164.61; *p* < 0.01; d = 0.97) and less confusion about the order of events (F = 150.95; *p* < 0.01; d = 0.90). The smallest difference between the fast group and the slow group is less future disorientation for the fast group compared to the slow group (F = 66.33; *p* < 0.01; d = 0.64). Effect sizes between the fast-and-slow group and the slow group are noteworthy enough, with e.g. less trauma for the fast-and-slow group (F = 50.39; *p* < 0.01; d = 0.39).

A similar pattern is present for temporal distance judgements, within which the largest difference for the short group compared to the long group is less confusion about the temporal order of events (F = 136.75; *p* < 0.01; d = 1.12), followed by less temporal rupture (F = 83.9; *p* < 0.01; d = 0.88) and less trauma (F = 69.94; *p* < 0.01; d = 0.80). The smallest difference between the short group and the long group is less future disorientation for the short group compared to the slow group (F = 21.89; *p* < 0.01; d = 0.47). Here again, effect sizes between the short-and-long group and the short group are noteworthy enough, with e.g. less confusion about the temporal order of events (F = 40.49; *p* < 0.01; d = 0.47). Interestingly, in both themes, those who had experienced fast or short time reported actually *more* social orientation, as well as an overall *easier* recall of events since the pandemic started (i.e. not just an absence of disorientation).

## Discussion

This study examined the connection between, on the one hand, passage of time and temporal distance judgements, and, on the other hand, various disruptions to social and temporal experience. These disruptions include the temporal order of events, future orientation, temporal self-location, temporal rupture, social disorientation, and psychological trauma. The data was collected in May and early June 2021, directly after the third national French lockdown and after more than 7 months of severe health restrictions in France starting in October 2021, including two national lockdowns and a superseding curfew which was about to end June 20, 2021. Therefore, the focus is not on the initial disruption caused by lockdowns per se but on the protracted psychological and experiential disturbances of the lengthy Covid-19 crisis and on people’s capacity to adjust to that prolonged crisis.

Our results confirm previous studies that found a slow passage of time to be more prevalent than a fast passage of time^1,5,9^, and longer temporal distances to be more prevalent than shorter temporal distances^9,13^. Our results also show bimodality for both PoT (Passage of Time) and TD (Temporal Distance), as found in previous studies^2,4,11,13^. In other words, while a slow passage of time was the more prevalent direction of disruption in PoT, a notable number of participants experienced a faster passage of time. Likewise, while longer temporal distance was the more prevalent TD disruption, a notable number of participants experienced shorter temporal distance instead. Having three separate questions for PoT and three separate questions for TD can help us clarify the emerging picture. Regarding PoT, 32.6% of participants described time as passing fast or very fast, compared with 42.2% of participants who described time as passing slowly or very slowly, when given a choice between time passing slowly or quickly overall (Q50). Nevertheless, looking at the other two questions, we see that a noteworthy number of participants (n = 247 out of a total sample of n = 3308) agree to both time passing slower and time passing faster *at times.* A similar effect is observed with respect to TD (n = 289). A substantial number of participants experienced disruptions in both directions of PoT and TD. This is an important finding, because it helps explain the bimodality found in previous research, as well as the differing directions of overall PoT distortions found in separate studies (e.g., slow in France^1^, Italy^7^, Brazil^3^ and Iraq^5^; and fast in Uruguay^8^ and Argentina^6^).

In the present study, we employed the concept of disorientation as a lens through which to examine the various disruptions experienced during the pandemic. A recent review by van Wassenhove^44^ on the links between isolation and temporal cognition advanced the idea that isolation disrupts the orientational mechanisms supporting temporal cognitive maps, resulting in a form of temporal disorientation. There is mounting evidence that the hippocampus encodes not only physical space but also temporal and social structures^45,46^. We can therefore talk of both temporal and social cognitive maps^45^, and by extension, of temporal and social orientation and disorientation. Researchers from different disciplines have used disorientation as a framework to analyse the psychological effects of the Covid-19 crisis. Here, we conceive of the disorientation experienced during the pandemic as an existential feeling that encompasses a variety of disruptions across affective, temporal and social dimensions^39,47^. Accordingly, we plotted the Future Orientation, Future Orientation, Temporal Self Location, Temporal Rupture, Social Disorientation and GPTS components on a spectrum from disoriented to oriented in order to observe their connection to both PoT (Passage of Time) and TD (Temporal Distance).

Previous work indicates an association between fast PoT (Passage of Time) and short TD (Temporal Distance)^4^. When we grouped participants according to their responses in PoT and TD and tested the average value of their responses for the questions in the other six components, our results pointed in a similar direction. Those in the slow passage of time group are consistently more disoriented than those in the fast passage of time group, and those in the long temporal distance group are consistently more disoriented than those in the short passage of time group. This is the case for all the components under study. Not only that, when we rank the temporal components from more disoriented to less disoriented, we obtain the same ranking for both the passage of time grouping and for the temporal distance grouping: Future Orientation, Temporal Rupture, Temporal Self Location, Future Orientation. This is the case whether we are ranking them based on the values for the slow PoT group, the fast PoT group, the long TD group, or the short TD group. This shows that, in terms of associated cognitive disruptions, the effect of slow passage of time is similar to the effect of long temporal distances, and also that the effect of fast passage of time is similar to the effect of short temporal distances. This double association is very visible in Fig. 2, where the similarity of the two plots is immediately apparent.

What is perhaps the most noteworthy finding of the present study is the difference between group 1 and group 2, that is, between participants experiencing a fast PoT (Passage of Time) and participants experiencing a slow PoT. Previous literature indicated that slow PoT was the more prevalent direction of disruption. Nevertheless, it was unclear whether slow PoT was also associated with stronger disturbances across the temporal and social domains, although associations between slow time on the one hand, and depression and anxiety on the other, were documented^2,8,16^. It might have been that (1) both slow and fast time resulted in equivalent disturbances, (2) slow and fast time resulted in different types of disturbances, (3) fast time was rarer but actually resulted in larger disturbances. However, what we found was that slow PoT—when compared to fast PoT—is not only more prevalent but is also associated with larger disturbances *in all the temporal components under study, as well as in social disorientation and trauma*. In contrast, participants in the fast PoT group only report clear disturbances during the pandemic when it comes to Future Orientation and to Temporal Rupture. Even more striking is that those participants who experienced solely a faster time reported being more socially oriented (i.e. less isolated, closer to others, etc.) than before the pandemic, and found it easier to remember the order of past events. For all components, the fast PoT group was “better off” than any of the other groups. It is better to experience no disruptions to one’s normal PoT than to experience a slower passage of time, but it is even better to experience faster than normal PoT. Of course, in the present study, we can only talk of association, not causation. It is likely that there is a common cause behind both fast PoT and the beneficial effects in all other components: an interesting candidate is that fast PoT might be a marker of the density of experience per standard temporal unit, itself conditioned by the dynamics of social interaction^48^. This view would be congruent with major results from Chaumon et al.^9^ on the fundamental role of perceived isolation on temporal experience during Covid-19 across countries.

One of the key questions that we wanted to address in our study concerned *temporal vertigo*—the sense of time both accelerating and decelerating or both contracting and dilating—which had been previously reported to be one of the features of the phenomenology of the pandemic^5,13,23,25^. To better understand temporal vertigo, we analysed the responses of those participants reporting both instances of slow and of fast PoT (group 3). This group represented 7% of our sample, for which we provide novel evidence as our design permitted conflicting time feelings to be expressed. One possibility was that *temporal vertigo* was in itself disorienting and therefore associated with other disruptions in the temporal and social domains. Instead, our findings indicate that the detrimental effect of slow PoT and the beneficial effect of fast PoT counteract each other. As a result, group 3 is always in between group 1 and group 2, as is clear in Fig. 2. In fact, while group 3 and group 4 tend to be close together, group 3 is always further along the disorientation-orientation axis. This indicates that the positive associations of fast PoT are large enough that those participants experiencing both slow and fast PoT are better off than those participants experiencing no disturbances on their PoT.

Turning the spotlight from PoT (*Passage of Time*) to TD (*Temporal Distance*) reveals a similar picture. *Short TD* (group 6) was associated with fewer disturbances, across all components, than the *both short and long TD* (group 7), which was associated with fewer disturbances than *neither short nor long TD* (group 8), which in turn was associated with fewer disturbances than *long TD* (group 5). The main notable difference with respect to the PoT groups analysis is that there is only one component, Future Orientation, for which participants experiencing only short TD report being more disoriented during than before the pandemic. Temporal Rupture, which was clearly on the negative side of the disoriented-oriented axis for all groups in the PoT comparison, is close to zero for group 6 in the TD comparison. Once again, these results indicate the association between fast-short and slow-long temporal distortions^2,4^. In both TD and PoT, temporal components are ranked in the same order (Future Orientation, Temporal Rupture, Temporal Self Location, Future Orientation). The same is true when considering Social Disorientation. The only exception to this mapping is GPTS, which for PoT is one position above Temporal Rupture and for TD is one position below Temporal Rupture. And as was the case with fast PoT, short TD was associated with an overall beneficial effect across all components.

While several previous studies focused on the initial impact of the pandemic, in particular on the first lockdown^7,10,11,16^, our data was collected during May and June 2021, a year and a half after the onset of the pandemic, and in the final weeks of the French curfew. As such, an important element of our study concerns our participants' adjustment to the pandemic conjecture. Within this context, our results indicate that time having regained a rapid pace is an important sign of overall well-being. However, some participants had not fully adjusted to the situation by the Spring of 2021, and remained, to differing extents, disoriented both temporally and socially. With these findings, the present study contributes to the literature on predictors of psychological distress during the Covid-19 crisis^3,49^. Our results reveal both PoT (*Passage of Time*) and TD (Temporal Distance) as key indicators of disorientation in a variety of domains.

There are several aspects that could be addressed in future research. The Covid-19 crisis has resulted in widespread disturbances in temporal experience, but it is also necessary to investigate how these relate exactly to other forms of psychological disturbances. For instance, autobiographical memory seems to influence passage of time judgements^27^, but so does perceived social isolation, which was identified as one of the most crucial experiential factors during the pandemic^9^. In turn, it remains unclear, for example, how memories relate to social disorientation in fostering temporal disruptions. And although our findings offer a clear picture for both PoT (*Passage of Time*) and TD (Temporal Distance), it remains uncertain whether similar patterns would appear with respondents not undergoing health restrictions. The pandemic brought under scrutiny ambiguous and conflicting aspects of time experience which had not been documented extensively in the literature beforehand. It would be interesting to ascertain whether the patterns that one can see in TD (Temporal Distance) and PoT, as well as in their relation to disorientation, hold true both in normal times and in more diverse forms of crises, such as wars or climatic catastrophes. In addition, it would be beneficial to investigate in more detail the causal connections behind the associations revealed in the present study. Once again, it is likely that there is a common cause behind fast PoT, short TD, and their associated beneficial effects.

All in all, our study reveals a complex dynamic behind the temporal, social and affective disturbances experienced during the Covid-19 crisis. There was a link between fast passage of time and short temporal distance judgements, and between slow passage of time and long distance judgements. More importantly, both slow passage of time and long distance judgements were associated with larger disturbances across all domains under study. These included temporal disruptions, such as the aptness to project oneself into the future, the sense of a rift between pre-pandemic and pandemic time, the ability to locate oneself in time, the capacity to recall the order of past events, as well as an overall sense of social disorientation, and trauma-specific disturbances. In contrast, both fast passage of time and short temporal distance judgements were associated with beneficial effects across all of the mentioned domains when compared not only to the slow/long time dyad but also to the experience of those participants who did not report any disturbance in neither passage of time nor temporal distance, and even both at once. This observation is novel in the literature and will be interesting for Covid-19 researchers as well as for time judgements researchers in general. In the context of public health policy, the present study suggests that decision makers should pay close attention to disruptions in time judgements and take the heterogeneity of these disruptions into account. Finally, our results indicate that the experience of slow passage of time is often associated with a host of temporal, social and affective disturbances, which can inform clinical interventions in times of crisis.

## Supplementary Information

Supplementary Information.

## Data Availability

We have made our dataset and analysis notebooks publicly available on https://www.kaggle.com/code/gurchani/testing-fast-slow-perception-of-time.

## References

[1] Droit-Volet S (2021). The persistence of slowed time experience during the COVID-19 pandemic: Two longitudinal studies in France. Front. Psychol..

[2] Ogden R (2021). Distortions to the passage of time during England’s second national lockdown: A role for depression. PLoS ONE.

[3] Cravo AM (2022). Time experience during social distancing: A longitudinal study during the first months of COVID-19 pandemic in Brazil. Sci. Adv..

[4] Kosak F, Schelhorn I, Wittmann M (2022). The subjective experience of time during the pandemic in Germany: The big slowdown. PLoS ONE.

[5] Alatrany SSJ, Ogden R, Falaiyah AM, ALdrraji HAS, Alatrany ASS (2022). The passage of time in Iraq during the Covid-19 pandemic. PLoS ONE.

[6] Brenlla ME, Germano G, Seivane MS, da Lama RF, Ogden R (2022). Experiences of distortions to the passage of time during the Argentinian Covid-19 pandemic. PLoS ONE.

[7] Cellini N, Canale N, Mioni G, Costa S (2020). Changes in sleep pattern, sense of time and digital media use during COVID-19 lockdown in Italy. J. Sleep Res..

[8] Loose T, Wittmann M, Vásquez-Echeverría A (2021). Disrupting times in the wake of the pandemic: Dispositional time attitudes, time perception and temporal focus. Time Soc..

[9] Chaumon M (2022). The Blursday database as a resource to study subjective temporalities during COVID-19. Nat. Hum. Behav..

[10] Droit-Volet S (2020). Time and Covid-19 stress in the lockdown situation: Time free, «Dying» of boredom and sadness. PLoS ONE.

[11] Ogden RS (2020). The passage of time during the UK Covid-19 lockdown. PLoS ONE.

[12] Wittmann M (2020). Subjective passage of time during the pandemic: Routine, boredom, and memory. KronoScope.

[13] Ogden RS, Piovesan A (2022). How long was it for you? Memories of the duration of the UK Covid-19 lockdown. PLoS ONE.

[14] Zauberman G, Levav J, Diehl K, Bhargave R (2010). 1995 Feels so close yet so far: The effect of event markers on subjective feelings of elapsed time. Psychol. Sci..

[15] Matthews WJ, Meck WH (2016). Temporal cognition: Connecting subjective time to perception, attention, and memory. Psychol. Bull..

[16] Martinelli N (2021). Time and emotion during lockdown and the Covid-19 epidemic: Determinants of our experience of time?. Front. Psychol..

[17] Pisano F (2021). A standardized prospective memory evaluation of the effects of COVID-19 confinement on young students. J. Clin. Med..

[18] Micillo, L. *et al. Time perspective predicts levels of anxiety and depression during the COVID-19 outbreak: A cross-cultural study*. (2021). 10.31234/osf.io/8tqap. 10.1371/journal.pone.0269396 PMC9521906 36174058

[19] Sobol M, Blachnio A, Przepiórka A (2020). Time of pandemic: Temporal perspectives related to compliance with public health regulations concerning the COVID-19 pandemic. Soc. Sci. Med..

[20] Barnes SJ (2021). Stuck in the past or living in the present? Temporal focus and the spread of COVID-19. Soc. Sci. Med..

[21] Holman EA, Grisham EL (2020). When time falls apart: The public health implications of distorted time perception in the age of COVID-19. Psychol. Trauma Theory Res. Pract. Policy.

[22] Wessels M (2022). Adapting to the pandemic: longitudinal effects of social restrictions on time perception and boredom during the Covid-19 pandemic in Germany. Sci. Rep..

[23] Velasco PF, Perroy B, Gurchani U, Casati R (2022). Lost in pandemic time: A phenomenological analysis of temporal disorientation during the Covid-19 crisis. Phenomenol. Cogn. Sci..

[24] Knight DM (2016). Temporal vertigo and time vortices on Greece’s central plain. Camb. J. Anthropol..

[25] Grondin S, Mendoza-Duran E, Rioux P-A (2020). Pandemic, quarantine, and psychological time. Front. Psychol..

[26] Wearden JH (2015). Passage of time judgements. Conscious. Cogn..

[27] Kosak F, Kuhbandner C, Hilbert S (2019). Time passes too fast? Then recall the past! Evidence for a reminiscence heuristic in passage of time judgments. Acta Psychol. (Amst.).

[28] Martinelli N, Droit-Volet S (2022). What factors underlie our experience of the passage of time? Theoretical consequences. Psychol. Res..

[29] Droit-Volet S, Wearden J (2016). Passage of time judgments are not duration judgments: Evidence from a study using experience sampling methodology. Front. Psychol..

[30] Proust J (2015). The representational structure of feelings. Open MIND.

[31] Wittmann M (2016). Felt Time: The Psychology of How We Perceive Time.

[32] Kent JN, Kilby CJ (2022). Predictors of psychological distress during self-isolation. Psychol. Psychother. Theory Res. Pract..

[33] Fernandez Velasco P, Gurchani U, Perroy B, Pelletreau-Duris T, Casati R (2022). Development and validation of a quantitative instrument for measuring temporal and social disorientation in the Covid-19 crisis. PLoS ONE.

[34] Kyriazos TA (2018). Applied psychometrics: The 3-faced construct validation method, a routine for evaluating a factor structure. Psychology.

[35] Olff M (2020). Screening for consequences of trauma: An update on the global collaboration on traumatic stress. Eur. J. Psychotraumatol..

[36] van Gils-Schmidt HJ, Verdonschot CP, Schaubroeck K (2020). Editorial ‘the value of disorientation’. Ethical Theory Moral Pract..

[37] Nelson JL (2020). Doubt, disorientation, and death in the plague time. Hastings Cent. Rep..

[38] Henk JVG-S (2020). Practical disorientation & transformative experience as a framework for understanding & exploring the Covid-19 pandemic’s impact. Rev. Filos. Apl..

[39] Ratcliffe M (2021). Disorientation, distrust and the pandemic. Glob. Discourse.

[40] di Friedberg MS (2021). Orientation, disorientation, reorientation: A reply to Fernández Velasco, Perroy and Casati. Glob. Discourse.

[41] Means AJ, Slater GB (2021). Collective disorientation in the pandemic conjuncture. Cult. Stud..

[42] Benedikter R, Fathi K (2022). Corona: The Once-in-a-Century Health Crisis and Its Teachings: Towards a more Multi-Resilient Post-Corona World.

[43] Oinas E, Heath M, Darkwah AK, Beoku-Betts J, Purkayastha B (2021). Disorientation, disbelief, distance. Global Feminist Autoethnographies During COVID-19.

[44] van Wassenhove V (2022). Temporal disorientations and distortions during isolation. Neurosci. Biobehav. Rev..

[45] Tavares RM (2015). A map for social navigation in the human brain. Neuron.

[46] Schafer M, Schiller D (2018). Navigating social space. Neuron.

[47] Fernandez Velasco P, Perroy B, Casati R (2021). The collective disorientation of the COVID-19 crisis. Glob. Discourse.

[48] Flaherty MG (2018). An S-shaped pattern in the perceived passage of time: how social interaction governs temporal experience. Lang. Cogn..

[49] Prout TA (2020). Identifying predictors of psychological distress during COVID-19: A machine learning approach. Front. Psychol..

