# Supplementary material for: Experiencing a slow passage of time was an indicator of social and temporal disorientation during the Covid-19 pandemic
Source: Sci Rep. 2022 Dec 26;12:22338. doi: 10.1038/s41598-022-25194-2 (PMC9792449; doi:10.1038/s41598-022-25194-2)
Supplement: Supplementary file 1 — Supplementary Information. [file 41598_2022_25194_MOESM1_ESM.pdf]

Appendix 1

We developed a quantitative Instrument for measuring Temporal and Social Disorientation (ITSD), aimed at major crises such as the Covid-19 pandemic. In this appendix we will elaborate on the characteristics of the sample and the validation process of this questionnaire.

Sample Characteristics

**Characteristics**	**Values**
Average Age	25.24
Median age	21.00
Number of Female Participants	2273
Number of Male Participants	990
Number of Participants that chose neither ‘male’ nor ‘female’	43
Average Socio-Economic Status (based on the MacArthur Scale of Subjective Social Status)	6.04
Number of students	2392
Number of workers	342

Number of retirees	64
Number of unemployed	25
Number of ‘chômage partiel’ (a French special status during the crisis, with wages mostly covered by the state and the person not actively working)	3
Average Number of Cohabitants	2.3

Table 1: Sample Characteristics of the 3306 participants having answered all questions

Exploratory Factor Analysis

We performed exploratory factor analysis and retained the factors with eigen-values more 1. Table 2 and Table 3 show the factor loadings for each component identified. Here we are listing the names of identified components and their short forms:

1. SD: Social Disorientation (Questions 20, 21, 22, 23, 24, 27)
2. PT: Passage of Time (Questions 48, 49, 50)
3. TOE: Temporal Order of Events (Questions 42, 43, 54)
4. TD: Temporal Distance (Questions 45, 46, 47)
5. FO: Future Orientation (Questions 52, 56, 57)
6. TSL: Temporal Self Location (Questions 40, 41)
7. ATO: Assisted Temporal Orientation (Questions 36, 37)

8. TR: Temporal Rupture (Questions 44, 58)

Items (questions)	PT	TOE	TD	FO	TSL	ATO	TR
**Section I**<br>Since the beginning of the Covid-19 crisis…							
Q36. I feel I'm more / less reliant on calendars or to-do lists to keep track of what I do.	-0.002	-0.050	-0.076	0.006	0.048	**0.992**	-0.054
Q37. I care more / less about following a routine (daily, or weekly).	0.033	0.019	0.017	0.050	-0.108	**0.397**	0.011
Q38. I feel that more / less activities or tasks would be doable in a day or in a week.	0.028	-0.007	0.021	0.239	-0.255	0.149	0.047
Q39. I feel more / less late overall on my commitments or my deadlines.	-0.047	-0.052	-0.040	-0.199	0.276	-0.077	-0.037
Q40. I get confused more / less often about which day of the week it is.	-0.059	-0.202	-0.067	-0.082	**0.431**	-0.012	-0.078
Q41. I get confused more / less often about which month of the year it is.	0.001	-0.141	-0.032	-0.033	**0.985**	0.038	-0.079

**Section II**							
Q42. At times, I feel confused about the order of events that occurred since the pandemic began.	0.001	**0.68**	0.060	0.040	-0.256	-0.027	0.152
Q43. At times, I feel confused about the order of events that occurred before the pandemic began.	0.006	**0.651**	0.033	-0.027	-0.045	-0.035	0.048
Q44. The period since the pandemic began feel connected / disconnected from the months and years prior.	0.047	0.072	0.155	0.139	-0.112	-0.042	**0.44**
Q45. At times, the beginning of the pandemic feels noticeably far away.	0.036	0.194	**0.674**	-0.012	-0.106	-0.075	0.180
Q46. At times, the beginning of the pandemic feels noticeably close.	-0.124	0.031	**0.676**	-0.082	-0.030	0.015	0.091
Q47. Overall, times before the pandemic feel as if they are further away / closer to me than they really are.	0.086	0.169	**0.547**	0.081	-0.090	-0.047	0.198
Q48. At times, since the pandemic began, time has been passing noticeably slowly.	**0.728**	0.067	0.087	0.000	-0.118	-0.028	0.151

Q49. At times, since the pandemic began, time has been passing noticeably quickly.	**0.697**	-0.060	0.081	0.091	0.063	0.056	-0.077
Q50. Overall, since the pandemic began, time has been passing slowly / quickly.	**0.828**	0.021	0.074	0.028	-0.052	-0.007	0.042
**Section III** Since the beginning of the Covid-19 crisis…							
Q52. I feel it is easier / harder to imagine for me to imagine the future.	0.054	0.119	0.074	**0.720**	-0.063	0.020	0.148
Q53. I feel it is easier / harder to imagine for me to recall events having taken place since the pandemic began.	-0.008	0.317	0.101	0.164	-0.214	-0.001	0.066
Q54. I feel it is easier / harder to imagine for me to recall events having taken place before the pandemic began.	0.003	0.397	0.138	0.126	-0.072	-0.021	0.028
Q55. I feel I ruminate more / less about past events emotionally negatively charged.	0.094	0.068	0.084	**0.327**	-0.106	-0.015	0.105
Q56. I feel I'm more / less anxious about my future.	0.060	0.084	0.092	**0.640**	-0.060	-0.029	0.140

Q57. I feel I'm more / less in control of my future.	0.059	0.102	0.060	**0.565**	-0.100	0.036	0.041
Q58. At times, the period since the pandemic began felt unreal to me.	0.043	0.139	0.094	0.084	-0.101	-0.059	**0.490**

Table 2: Factor Loadings for Temporal Disorientation Components. Questions were asked in French (see the full original questionnaire in the supplementary materials); underlined are adverbs used as a Likert-scale set of 5 answers; boldened factor loadings indicate questions used for the component in both solution 2 and solution 3.

**Items (questions)**	**Social Disorientation (SD)**
Since the beginning of the Covid-19 crisis…	
Q20. I lost contact with people I was close to before.	**0.65**
Q21. I feel less able to find someone to turn to.	**0.67**
Q22. I feel more isolated from others.	**0.66**
Q23. I am more unsure of my true membership in certain social groups that I previously felt fully part of.	**0.65**
Q24. I am less sure I am close to the people I used to feel close to.	**0.82**

Q25. I was able to build and maintain new relationships through digital tools.	0.217
Q26. I'm less sure of my closeness to the people I interact with primarily through digital tools.	-0.422
Q27. I feel like my social universe has shrunk.	**0.60**

Table 3: Factor Loadings for Social Disorientation Component. Questions were asked in French (see the full original questionnaire in the supplementary materials); questions were asked with a Likert-scale set of 5 possible answers; boldened factor loadings indicate questions used for the SD in both solution 2 and solution 3.

Reliability

To establish the internal consistency, we calculated Cronbach's alpha values for each of the components detected in EFA. Table 4 shows the result of these tests.

**Construct**	**Cronbach's alpha value**
**Social Disorientation Factor**	
Social Disorientation (SD)	0.865
**Temporal Disorientation Factors**	
Passage of time (PT)	0.80
Temporal Order of Events (TOE)	0.63

Temporal Distance (TD)	0.65
Future Orientation (FO)	0.74
Temporal Self Location (TSL)	0.65
Assisted Temporal Orientation (ATO)	0.52

Appendix 2

			Temporal Distance			
	Time-Long and Time-Short-&-Long	Time-Long and Time-Short	Time-Long and Nor-Short-nor-Long	Time-Short and Nor-Short-nor-Long	Time-Short and Time-Short-&-Long	Time-Short-&-Long and Nor-Short-nor-Long
**Social Disorientation**	[9.11, 0.0, 0.223]	[50.08, 0.0, -0.673]	[0.55, 0.46, -0.037]	[55.38, 0.0, 0.635]	[19.55, 0.0, 0.459]	[8.93, 0.0, 0.185]
**Global Psychotrauma**	[14.29, 0.0, 0.278]	[69.94, 0.0, -0.796]	[2.02, 0.16, -0.071]	[69.5, 0.0, 0.72]	[24.28, 0.0, 0.516]	[11.11, 0.0, 0.205]
**Temporal Rupture**	[9.72, 0.0, 0.23]	[83.9, 0.0, -0.878]	[1.04, 0.31, -0.05]	[92.39, 0.0, 0.828]	[38.64, 0.0, 0.65]	[8.5, 0.0, 0.18]
**Temporal Self-Location**	[8.83, 0.0, 0.22]	[25.79, 0.0, -0.535]	[6.46, 0.01, -0.123]	[18.6, 0.0, 0.418]	[6.93, 0.01, 0.292]	[2.92, 0.09, 0.104]
**Future Orientation**	[4.26, 0.04, 0.153]	[21.89, 0.0, -0.469]	[1.72, 0.19, 0.065]	[34.17, 0.0, 0.536]	[8.53, 0.0, 0.313]	[12.37, 0.0, 0.218]
**Temporal Distance**	[277.99, 0.0, 1.103]	[3.51, 0.06, -0.202]	[1003.39, 0.0, 1.938]	[285.22, 0.0, 2.062]	[71.92, 0.0, -1.045]	[1278.33, 0.0, 2.292]
**Temporal Order of Events**	[40.49, 0.0, 0.466]	[136.75, 0.0, -1.12]	[18.29, 0.0, -0.215]	[103.27, 0.0, 0.894]	[36.71, 0.0, 0.64]	[16.45, 0.0, 0.249]
**Passage of Time**	[5.09, 0.02, 0.164]	[3.8, 0.05, -0.193]	[20.01, 0.0, 0.225]	[19.84, 0.0, 0.412]	[0.02, 0.89, 0.015]	[38.1, 0.0, 0.367]
	**Table Description:** Each cell contains these 3 values in the following order [f-stats, p-value, cohen's d]					

Mean for components, per temporal distance group

Component	Time felt longer	Time felt neither long nor short	Time felt both long and short	Time felt short
Temporal Order of Events	-0.32	-0.11	0.14	0.82
Social Disorientation	-0.32	-0.30	-0.16	0.20
Temporal Self Location	-0.42	-0.33	-0.26	-0.06
Temporal Rupture	-0.82	-0.78	-0.64	-0.09
Global Psycho Trauma	-0.57	-0.52	-0.42	-0.27
Future Orientation	-0.77	-0.83	-0.65	-0.41

Appendix 3

Passage Of Time

	Time-Fast and Time-Slow-&-Fast	Time-Fast and Time-Slow	Time-Fast and Nor-Slow-nor-fast	Time-Slow and Nor-Slow-nor-fast	Time-Slow and Time-Slow-&-Fast	Time-Slow-&-Fast and Nor-Slow-nor-fast
**Social Disorientation**	[59.81, 0.0, -0.572]	[164.61, 0.0, 0.974]	[152.8, 0.0, 0.856]	[3.51, 0.06, -0.092]	[44.95, 0.0, -0.368]	[39.92, 0.0, 0.27]
**Global Psychotrauma**	[108.19, 0.0, -0.771]	[261.2, 0.0, 1.22]	[190.07, 0.0, 0.959]	[17.87, 0.0, -0.208]	[50.39, 0.0, -0.391]	[16.93, 0.0, 0.176]
**Temporal Rupture**	[39.38, 0.0, -0.464]	[99.98, 0.0, 0.762]	[81.2, 0.0, 0.616]	[9.31, 0.0, -0.147]	[25.42, 0.0, -0.276]	[9.89, 0.0, 0.134]
**Assisted Temporal Orientation**	[0.59, 0.44, 0.061]	[12.12, 0.0, -0.282]	[0.36, 0.55, -0.045]	[19.89, 0.0, 0.212]	[11.79, 0.0, 0.188]	[0.15, 0.7, 0.016]
**Temporal Self-Location Orientat**	[25.6, 0.0, -0.398]	[88.67, 0.0, 0.744]	[46.06, 0.0, 0.488]	[27.63, 0.0, -0.251]	[23.0, 0.0, -0.265]	[0.78, 0.38, 0.037]
**Future Orientation**	[39.97, 0.0, -0.489]	[66.33, 0.0, 0.639]	[82.27, 0.0, 0.652]	[0.03, 0.87, 0.008]	[4.24, 0.04, -0.113]	[8.23, 0.0, 0.121]
**Temporal Distance**	[9.53, 0.0, -0.238]	[23.46, 0.0, 0.377]	[55.56, 0.0, 0.533]	[9.61, 0.0, 0.15]	[4.05, 0.04, -0.111]	[35.65, 0.0, 0.252]
**Temporal Order of Events**	[60.32, 0.0, -0.552]	[150.95, 0.0, 0.903]	[83.12, 0.0, 0.595]	[40.77, 0.0, -0.312]	[40.18, 0.0, -0.348]	[0.83, 0.36, 0.039]
**Passage of Time**	[330.3, 0.0, 1.431]	[435.42, 0.0, 1.522]	[653.73, 0.0, 2.042]	[402.6, 0.0, 1.109]	[2319.82, 0.0, -2.733]	[4356.04, 0.0, 2.939]

Table Description: Each cell contains these 3 values in the following order [f-stats, p-value, cohen's d]

Mean for components, per time passage group

Component	Time passed slower	Time felt neither slow nor fast	Time felt both slow and fast	Time passed faster
Temporal Order of Events	-0.39	-0.09	-0.05	0.54
Social Disorientation	-0.42	-0.35	-0.15	0.26
Temporal Self-Location Orientation	-0.49	-0.32	-0.30	-0.03
Global Psychotrauma	-0.63	-0.54	-0.46	-0.20
Temporal Rupture	-0.90	-0.79	-0.68	-0.32
Future Orientation	-0.84	-0.85	-0.75	-0.38

Appendix 4

Quantitative questionnaire

**English Version**	**French Version**
Living the Pandemic. Qualitative questionnaire<br><br>We invite you to participate in a study. The purpose of this questionnaire is to better understand the psychological effects of the Covid-19 health crisis. We will ask you some questions about different aspects of your experience during the Covid-19 crisis. This research is part of the Dis-Covid project, hosted at the Institut Jean Nicod (ENS, EHESS, CNRS) and funded by the Agence Nationale de la Recherche (ANR). Participation in the project requires your informed consent. Before proceeding, please	Vivre la pandémie. Questionnaire qualitatif<br><br>Nous vous invitons à participer à une étude. Le but de ce questionnaire est de mieux comprendre les effets psychologiques de la crise sanitaire du Covid-19. Nous vous poserons quelques questions sur différents aspects de votre expérience pendant la crise de Covid-19. Cette recherche fait partie du projet Dis-Covid, hébergé à l'Institut Jean Nicod (ENS, EHESS, CNRS) et financé par l'Agence Nationale de la Recherche (ANR). La participation au projet nécessite votre consentement éclairé. Avant de poursuivre, veuillez prendre en compte les informations

consider the following information:<br><br>The survey will take 10-20 minutes to complete.<br>There are no risks or benefits of any kind associated with this study.<br>Your privacy will be maintained in all published and written data resulting from the study.<br>Participation in this research study is voluntary.<br>At any time, you may refuse to participate in the study without penalty.<br><br>By proceeding with the study, you certify that you have read this form and have agreed to participate in accordance with the above conditions.<br><br>If you have any questions or comments, please contact us at: pfernandezvelasco@ens.fr"<br><br>Translated with www.DeepL.com/Translator (free version)	suivantes :<br><br>L'enquête prendra de 10 à 20 minutes à remplir.<br>Cette étude ne comporte aucun risque ou avantage de quelque nature que ce soit.<br>Votre vie privée sera préservée dans toutes les données publiées et écrites résultant de l'étude.<br>La participation à cette étude de recherche est volontaire.<br>À tout moment, vous pouvez refuser de participer à l'étude sans aucune pénalité.<br><br>En procédant à l'étude, vous certifiez que vous avez lu ce formulaire et que vous avez accepté de participer conformément aux conditions susmentionnées.<br><br>Si vous avez des questions ou des commentaires, veuillez nous contacter à l'adresse suivante : pfernandezvelasco@ens.fr
Age	Age
Gender	Sexe
Location (postal code, country)	Localisation (code postal, pays)
Number of family members in your home	Nombre de membres de votre famille dans votre habitation
Number of people in your home who are not members of your family	Nombre de personnes dans votre habitation qui ne sont pas membres de votre famille
Professional situation	Situation professionnelle
Have you tested positive for Covid-19 in the past six months?	Avez-vous été testé(e) positif(ve) au Covid-19 au cours des six derniers mois ?
Have you been vaccinated against Covid-19?	Avez-vous été vacciné contre le Covid-19 ?
Do you have a medical condition (e.g. diabetes) that puts you at increased risk of hospitalization if you were to get Covid-19?	Avez-vous une maladie (par exemple le diabète) qui vous expose à un risque accru d'hospitalisation si vous deviez attraper le Covid-19 ?
Now imagine that the scale below represents the position of people in our society. At the top of the scale are the richest people, the best educated, and those with the best jobs. At the bottom of the scale are people with the least	Imaginez maintenant que l'échelle ci-dessous représente la position des gens dans notre société. Au sommet de l'échelle se trouvent les gens les plus riches, les mieux éduqués, et ceux qui ont les meilleurs emplois. Au bas de

money, the lowest level of education, and little or no employment. On this axis, you are asked to choose where you think your parents are located (one choice only).	l’échelle se trouvent les gens ayant le moins d’argent, le plus faible niveau d’éducation, et des emplois pas intéressants voire pas d’emploi du tout. Sur cet axe, il vous est demandé de choisir l’endroit où vous pensez que vos parents se trouvent (un seul choix).
Before the Covid-19 crisis...	Avant la crise de la Covid-19...
How many minutes of physical activity did you do on average each week (for example, biking or jogging)?	Combien de minutes d'activité physique pratiquiez-vous en moyenne chaque semaine (par exemple, du vélo ou du jogging) ?
On average, how much time did you spend at home during a weekday (Monday-Friday)?	Combien de temps passiez-vous en moyenne à la maison pendant un jour de semaine (lundi-vendredi) ?
How much time did you spend on average at home on a weekend day (Saturday, Sunday)?	Combien de temps passiez-vous en moyenne à la maison pendant un jour de week-end (samedi, dimanche) ?
In the last month...	Au cours du dernier mois…
How many minutes of physical activity did you engage in on average each week (e.g., biking or jogging)?	Combien de minutes d'activité physique avez-vous pratiqué en moyenne chaque semaine (par exemple du vélo ou du jogging) ?
How much time did you spend on average at home during a weekday (Monday-Friday)?	Combien de temps avez-vous passé en moyenne à la maison pendant un jour de semaine (lundi-vendredi) ?
How much time did you spend on average at home on a weekend day (Saturday, Sunday)?	Combien de temps avez-vous passé en moyenne à la maison pendant un jour de week-end (samedi, dimanche) ?
Since the beginning of the Covid-19 crisis...	Depuis le début de la crise de la Covid-19...
Do you keep a diary in which you write at least once a week?	Tenez-vous un journal intime dans lequel vous écrivez au moins une fois par semaine ?
Since the beginning of the pandemic, has your schedule become more or less stable? (For example: do you wake up at the same time every day? Do you do the same type of activity from one day to the next? etc.)	Depuis le début de la pandémie, votre emploi du temps est-il devenu plus ou moins stable ? (Par exemple : vous réveillez-vous tous les jours à la même heure ? Faites-vous le même type d'activité d'un jour à l'autre ? etc…)
Since the beginning of the covid-19 crisis...	Depuis le début de la crise de la covid-19...
I lost contact with people I was close to before.	J'ai perdu le contact avec des personnes dont j'étais proche auparavant.
I feel less able to find someone to turn to.	Je me sens moins capable de trouver quelqu'un vers qui me tourner.
I feel more isolated from others.	Je me sens plus isolé des autres.
I am more unsure of my true membership in certain social groups that I previously felt fully part of.	Je suis plus incertain(e) de mon appartenance réelle à certains groupes sociaux dont je me sentais faire pleinement partie auparavant.

I am less sure I am close to the people I used to feel close to.	Je suis moins sûr(e) d'être proche des personnes dont je me sentais proche auparavant.
I was able to build and maintain new relationships through digital tools.	J'ai pu construire et entretenir de nouvelles relations grâce aux outils numériques.
I'm less sure of my closeness to the people I interact with primarily through digital tools.	Je suis moins sûr de ma proximité avec les personnes avec lesquelles j'interagis principalement par des outils numériques.
I feel like my social universe has shrunk.	J'ai l'impression que mon univers social s'est rétréci.
Since the beginning of the Covid-19 crisis…	Depuis le début de la crise de la covid-19...
I feel I'm more / less reliant on calendars or to-do lists to keep track of what I do.	J'ai l'impression d'être plus / moins dépendant de calendriers, ou de listes de tâches, ou un journal, pour suivre ce que je fais.
I care more / less about following a routine (daily, or weekly).	Je me soucie plus / moins de suivre une routine dans mon quotidien (chaque jour, ou chaque semaine).
I feel that more / less activities or tasks would be doable in a day or in a week.	J'ai le sentiment que plus / moins d'activités ou de tâches sont réalisables dans une journée ou une semaine.
I feel more / less late overall on my commitments or my deadlines.	Je me sens plus / moins en retard en général sur tous mes engagements ou mes échéances.
I get confused more / less often about which day of the week it is.	Je suis confus plus / moins souvent quand j'essaie de savoir quel jour de la semaine il est.
I get confused more / less often about which month of the year it is.	Je suis confus plus / moins souvent quand j'essaie de savoir quel mois de l'année il est.
At times, I feel confused about the order of events that occurred since the pandemic began.	Par moments, l'ordre des événements survenus depuis le début de la pandémie me paraît confus.
At times, I feel confused about the order of events that occurred before the pandemic began.	Par moments, l'ordre des événements survenus avant le début de la pandémie me paraît confus.
The period since the pandemic began feel connected / disconnected from the months and years prior.	La période depuis le début de la crise du Covid-19 me semble connectée / déconnectée avec les mois et les années précédentes.
At times, the beginning of the pandemic feels noticeably far away.	Par moments, le début de la pandémie me semble sensiblement lointain.
At times, the beginning of the pandemic feels noticeably close.	Par moments, le début de la pandémie me semble sensiblement proche.
Overall, times before the pandemic feel as if they are further away / closer to me than they really are.	Dans l'ensemble, la période précédant la pandémie me parait être plus lointaine / plus proche que ce qu'elle l'est réellement.
At times, since the pandemic began, time has been passing noticeably slowly.	Par moments, depuis le début de la pandémie, le temps passe sensiblement lentement.

At times, since the pandemic began, time has been passing noticeably quickly.	Par moments, depuis le début de la pandémie, le temps passe sensiblement vite.
Overall, since the pandemic began, time has been passing slowly / quickly.	Dans l'ensemble, depuis le début de la pandémie, le temps s'est écoulé lentement / rapidement.
Since the beginning of the Covid-19 crisis…	Depuis le début de la crise de la covid-19...
I feel it is easier / harder to imagine for me to imagine the future.	J'ai l'impression qu'il est plus plus facile / difficile pour moi d'imaginer le futur.
I feel it is easier / harder to imagine for me to recall events having taken place since the pandemic began.	J'ai l'impression qu'il est plus facile / difficile pour moi de se rappeler des événements ayant eu lieu depuis le début de la crise de la Covid-19.
I feel it is easier / harder to imagine for me to recall events having taken place before the pandemic began.	J'ai l'impression qu'il est plus facile / difficile pour moi de se rappeler des événements ayant eu lieu avant le début de la crise de la Covid-19).
I feel I ruminate more / less about past events emotionally negatively charged.	J'ai l'impression de ruminer plus / moins souvent à propos d'événements passés émotionnellement négatifs.
I feel I'm more / less anxious about my future.	J'ai l'impression d'être plus / moins anxieux quant à mon avenir.
I feel I'm more / less in control of my future.	J'ai l'impression d'être plus / moins en contrôle de mon avenir.
At times, the period since the pandemic began felt unreal to me.	Par moments, la période depuis le début de la crise de la Covid-19 m'a paru irréelle.
In the past month, compared to before the Covid-19 crisis, have you...	Au cours du dernier mois, par rapport à avant la crise de la Covid-19, avez-vous…
... had the impression of being easily destabilized?	... eu l'impression d'être facilement déstabilisé ?
... felt detached from people, activities or your environment?	... eu l'impression d'être détaché des gens, des activités ou de votre environnement ?
... tended to feel useless?	... eu tendance à vous sentir inutile ?
... had outbursts of anger that you couldn't control?	... eu des accès de colère que vous ne pouviez pas contrôler ?
... felt anxiety?	... ressenti de l'anxiété ?
...were unable to stop worrying?	... été incapable d'arrêter de vous inquiéter ?
... been depressed, down or discouraged?	... été déprimé, abattu ou découragé ?
...experienced little interest or pleasure in doing things?	... éprouvé peu d'intérêt ou de plaisir à faire des choses ?
... had problems falling or staying asleep?	... eu des problèmes pour s'endormir ou rester endormi ?
...tried to hurt yourself on purpose?	... essayé de vous blesser intentionnellement ?
... perceived or experienced the world or other people differently, so that things seem dreamlike, strange, or unreal?	... perçu ou expérimenté le monde ou les autres personnes différemment, de sorte que

	les choses semblent comme dans un rêve, étranges, ou irréelles ?
... felt detached or separate from your body (e.g., the feeling of looking down on yourself or being an outside observer of your own body)?	... eu l'impression d'être détaché ou séparé de votre corps (par exemple, l'impression de vous regarder d'en haut ou d'être un observateur extérieur de votre propre corps) ?
Thank you very much for completing the survey. To submit it, please click below.	Merci beaucoup d'avoir répondu au questionnaire. Pour le soumettre, veuillez cliquer ci-dessous.

Qualitative Questionnaire

One of the chief features of the Covid-19 crisis is that we find ourselves in a shifting landscape. The resulting disorientation extends into many domains of our individual and collective lives. Many people feel temporally, socially or politically disoriented. The Dis-Covid project, funded by the ANR, aims at collecting subjective reports about people's experience during the Covid-19 crisis to gain insight into the social, psychological and political dynamics of the current pandemic.

1. Many people have been feeling time distortions as a result of the Covid-19 health crisis. For instance, you may have felt a change in how quickly or slowly time passes, you may have found it difficult to project yourself into the future or to make plans, you may have been unsure of what day of the week it was, or you may feel that past events are further (or nearer) into the past than they actually are, to name but a few examples. Please use the space below to narrate any ways in which you might have felt time distortions as a result of the current health crisis. Feel free to write down anything you feel is relevant (e.g. an episode that struck you particularly).

2. Many people have been feeling socially out of place as a result of the Covid-19 health crisis. For instance, you may have felt unsure whether you still truly belong to social groups (whether personal or professional) whose interactions have had to adapt to social distancing, you may have felt unusually awkward in social situations, you might have felt confused regarding the degree of physical closeness to maintain with a friend or relative, or you may have felt lonely yet incapable of reaching out to other people, to name but a few examples. Please use the space below to outline any ways in which you might have felt socially out of place as a result of the current health crisis. Feel free to write down anything you feel is relevant (e.g. an episode that struck you particularly).

3. Many people have been feeling at a loss when trying to navigate the information related to the Covid-19 health crisis. For instance, you might have encountered difficulties to find your way in the current information environment, you might feel it is hard to assess the reliability and the relevance of the news you find in social media, you might feel like the frames of reference you once used to understand politics are no longer useful, or you might feel that established experts are not helpful guides in the current pandemic, to name but a few examples. Please use the space below to outline any ways in which you might have felt at a loss when trying to navigate the information related to the Covid-19 health crisis. Feel free to write down anything you feel is relevant (e.g. an episode that struck you particularly).

Appendix 5

Means for component, per group
